# Improving Detection of Prediabetes in Children and Adults: Using Combinations of Blood Glucose Tests

**DOI:** 10.3389/fpubh.2015.00260

**Published:** 2015-11-20

**Authors:** Ike S. Okosun, J. Paul Seale, Rodney Lyn, Y. Monique Davis-Smith

**Affiliations:** ^1^Division of Epidemiology and Biostatistics, School of Public Health, Georgia State University, Atlanta, GA, USA; ^2^Department of Family Medicine, Mercer University School of Medicine, Macon, GA, USA; ^3^Division of Health Management and Policy, School of Public Health, Georgia State University, Atlanta, GA, USA

**Keywords:** glycated hemoglobin, glycemia, blood glucose tests, prediabetes

## Abstract

**Highlights:**

- The combined use of fasting plasma glucose and hemoglobin A1c test is associated with significantly higher diagnostic rates of prediabetes across age, race/ethnicity, and BMI than using only one test.
- Combined use of fasting plasma glucose, hemoglobin A1c, and oral glucose tolerance test do not improve the overall and gender-specific prediabetes prevalence beyond what is observed using a combination fasting plasma glucose and hemoglobin A1c test.
- A redefined hemoglobin A1c test that incorporates racial/ethnic, gender, age, and BMI differences may provide a better way to use hemoglobin A1c test in population-based and clinical settings.

**Aim:**

To determine combinations of blood glucose tests: oral glucose tolerance (OGT), fasting plasma glucose (FPG), and hemoglobin A1C (HbA1C) that are associated with highest diagnostic rates of prediabetes in non-diabetic American children and adults.

**Methods:**

The 2007–2008 U.S. National Health and Nutrition Examination Surveys data were used for this study. Overall and specific prevalence of prediabetes (defined using OGT + FPG, OGT + HbA1C, HbA1C + FPG, and OGT + FPG + HbA1C tests) were determined across age, race/ethnicity, sex, and BMI categories.

**Results:**

FPG + HbA1C test was associated with significantly higher diagnostic rates of prediabetes across age, race/ethnicity, and BMI. Estimates of overall prevalence of prediabetes using OGT + FPG, OGT + HbA1C, HbA1C + FPG, and OGT + FPG + HbA1C tests were 20.3, 24.2, 33, and 34.3%, respectively. Compared to OGT + FPG, the use of HbA1C + FPG test in screening was associated with 44.8, 135, 38.6, and 35.9% increased prevalence of prediabetes in non-Hispanic White, non-Hispanic Black, Mexican-American, and other racial/ethnic men, respectively. The corresponding values in women were 67.8, 140, 37.2, and 42.6%, respectively. Combined use of all blood glucose tests did not improve the overall and gender-specific prediabetes prevalence beyond what was observed using HbA1C + FPG test.

**Conclusion:**

HbA1C criteria were associated with higher diagnosis rates of prediabetes than FPG and OGT tests in non-diabetic American children and adults. Using a combination of HbA1C and FPG test in screening for prediabetes reduces intrinsic systematic bias in using just HbA1C testing and offers the benefits of each test. A well-defined HbA1C that takes into consideration race/ethnicity, gender, age, and body mass index may improve detection of prediabetes in population and clinical settings.

## Introduction

Approximately, 24 million Americans are suffering from type 2 diabetes while more than 65 million others have prediabetes (1, 2). The projected number of Americans with diagnosed type 2 diabetes is 39.0 million for 2050 (3), and a 100% increase in prediabetes has been predicted for 2030 (1). Hence, accurate identification of people with prediabetes is imperative before applications of pharmacological and lifestyle interventions for the prevention and delay of type 2 diabetes. The application of a screening test that is both robust and handy is, therefore, vital for promptly ascertaining subjects with prediabetes. A robust and quick prediabetes diagnostic test can reduce overall societal costs of diabetes by motivating subjects with prediabetes to seek diabetes preventative care.

Fasting plasma glucose (FPG) and oral glucose tolerance (OGT) tests are greatly used in screening and diagnosing type 2 diabetes. While the OGT test is mostly recognized as the benchmark for diagnosing type 2 diabetes, the test is laborious and uncomfortable to patients. On the other hand, FPG is preferred over OGT test since it is simple to conduct and the results are highly reproducible. Applying FPG exclusive of OGT testing often underestimates the prevalence of type 2 diabetes because the test often miss many persons who have elevated OGT as well as normal FPG (4). The American Diabetes Association (ADA) supports the use of hemoglobin A1C (HbA1C) test in identifying subjects with type 2 diabetes (5). ADA also advocates HbA1C test in identifying at-risk persons and supports its use for establishing association between elevated HbA1C and microvascular diseases (5). HbA1C is an integrated measure of circulating glucose levels, providing an overall appraisal of glucose level in the last 60–90 days, and is a benchmark for prospective analysis of glycemic homeostasis (5).

Elevated HbA1C in non-diabetic adults is associated with incident diabetes, cardiovascular disease morbidity and overall-mortality (6–8). Unlike OGT, HbA1C test is quick and convenient and can be completed any time notwithstanding duration of fasting nor the type of prior meal. There are two important limitations to the use HbA1C test in identifying subjects with type diabetes. One, HbA1C test has good specificity for elevated glucose only at HbA1C of 6.5% or greater (9, 10), and not very sensitive at <6.5% levels. Lack of good sensitivity for blood glucose at HbA1C of <6.5% may be due to inter-individual variations that are related to red cell penetration, glycation, hemoglobin species, vitamin and medication status, and red cell half-life, as well as hyperglycemia (9–12). Two, there are important racial/ethnic differences in HbA1C level. At any glycemic level, Blacks present with much higher HbA1C values compared to Whites (13–18). Hence, there is an ongoing debate on the appropriateness of using the same HbA1C cutoff points in the diagnosis of type 2 diabetes in Blacks and Whites (19, 20).

Thus far, there has been limited discussion regarding the accuracy of HbA1C test in diagnosing prediabetes. The aim of this study is to determine combinations of blood glucose tests that are associated with highest prevalence of prediabetes in American children and adults. The result of this study will provide crucial insight into how to improve detection of prediabetes giving its increasing prevalence in children and adults. If specific population groups can be identified in which accuracy of HbA1C test is decreased, confirmatory testing with more accurate tests, such as FPG and OGT tests, might be advantageous in identifying individuals with truly impaired glucose tolerance (IGT) who are at risk for future disease. We hypothesize that the combinations of blood glucose tests that are associated with highest prevalence of prediabetes will vary across age, gender, sex, race/ethnicity, and BMI categories in American children and adults.

## Materials and Methods

### Subjects and Study Design

The U.S. National Center for Health Statistics (NCHS) provided the 2007–2008 National Health and Nutrition Examination Surveys (NHANES) data that were utilized for this study. NHANES are complex sampling designs that were administered to a representative sample of U.S. non-institutionalized civilians. In the surveys, study subjects were interviewed in their homes and laboratory and physical examination were done in mobile examination centers for limited number of participants. The survey plans have been described by other investigators (21, 22) and are published in the World Wide Web (23). NCHS institutional review board approved all NHANES study protocols. Informed consents were obtained from all adult participants. For children who were <18 years old children, informed consents were obtained from their legal guardians.

For this study, only participants (ages 12–75 years) with no missing values for age, height, weight, and assayed for OGT, FPG and HbA1C were investigated. In NHANES, anthropometric measures were obtained using established protocols (23). Height was measured with a fixed stadiometer and weight was determined using a Toledo digital scale (Seritex, Carlstadt, NJ, USA). BMI and race/ethnicity were also included among the variables for this study. In NHANES, participants who were 12 years and older were tested for abnormal blood glucose values using FPG, OGT, and HbA1C tests (23).

### Definition of Terms

This study was restricted to non-diabetic children and adults (ages 12–75 years). In this study, subjects were categorized as non-diabetic if they had FPG of <125 mg/dl or FPG of <199 mg/dl on a 2-h OGT test and HbA1C <6.5% (4, 5). Prediabetes comprises impaired fasting glucose (IFG) expressed as FPG 100–125 mg/dl, IGT as OGT 140–199 mg/dl, and HbA1C 5.7–6.4% (4, 5). Two hundred and forty-two participants with history of diabetes and 57 pregnant women were disqualified in this analysis. Participants with congestive heart failure, coronary heart disease, angina/angina pectoris, heart attack, and stroke were also excluded in this study. We consider these diseases to be associated with glycemia (24). Subjects who were eligible in this study were not different from those who were excluded in terms of age, gender, BMI, and race/ethnicity. In this study, four racial/ethnic groups consisting of non-Hispanic Whites, non-Hispanic Blacks, Mexican-Americans, and others (Hispanics and multiracial groups) were used. BMI was categorized as low body weight, healthy weight, overweight, class I obesity, class II obesity and morbidly obese based on BMI of <20, 20–24.9, 25–29.9, 30–34.9, 35–39.9, and >40 kg/m^2^, respectively (25).

### Statistical Analysis

SAS for Windows (SAS Release 9.1) was the statistical package used in this analysis. To account for uneven sampling probabilities, oversampling, and non-response, we used appropriate sample weights for the analyses. We estimated SEs using the SUDAAN statistical program (26). Racial/ethnic differences in continuous variables, including age, anthropometric and glycemic variables were determined with one-way analysis of variance (ANOVA). Racial/ethnic and sex-differences in prevalence of prediabetes using OGT + FPG, OGT + HbA1C, HbA1C + FPG, and OGT + FPG + HbA1C tests were determined using the χ^2^ tests. Pearson χ^2^ and Tukey’s *post hoc* tests were used for pairwise comparisons for categorical and continuous variables, respectively. The relationships between HbA1C, OGT, and FPG were determined using age and BMI adjusted Pearson’s product-moment correlation coefficients. Prevalences of prediabetes were determined using HbA1C, OGT, and FPG definitions of prediabetes (4, 5). Overall and specific prevalence of prediabetes (defined using OGT + FPG, OGT + HbA1C, HbA1C + FPG, and OGT + FPG + HbA1C) were also determined across age, sex, race/ethnicity, and BMI categories. In this investigation, *P* < 0.05 and 95% confidence intervals were computed and used to establish statistical significance.

## Results

### Characteristics of Studied Population

The basic anthropometric and clinical characteristics of non-Hispanic Whites, non-Hispanic Blacks, Mexican-Americans, and racial/ethnic groups that were eligible for this study are shown in Table 1. There were racial/ethnic differences in age, anthropometric, and glycemic variables. Non-Hispanic Whites were older and taller than non-Hispanic Blacks, Mexican-Americans and other racial/ethnic groups (*P* < 0.001). Defined using BMI, non-Hispanic Blacks were heavier as compared to non-Hispanic Whites and Mexican-Americans (*P* < 0.001). Non-Hispanic Blacks also had higher values of HbA1C and lower prevalence of OGT and FPG compared to non-Hispanic Whites and Mexican-Americans (*P* < 0.01). The proportion of non-Hispanic Blacks with HbA1C values of 5.7–6.4% was higher than those of non-Hispanic Whites, Mexican-Americans and Other racial/ethnic groups (*P* < 0.001). Fewer non-Hispanic Blacks had IFG as compared with other racial/ethnic groups (*P* < 0.001).

**Table 1:** **Anthropometric and clinical characteristics of studied populations of Americans who were eligible for this study**.

Variables *n*	Non-Hispanic White 2714	Non-Hispanic Black 1290	Mexican-American 1141	Others 1004	*P*-value
Age (years)	46.7^a^ ± 0.41	38.7^b^ ± 0.56	35.7^c^ ± 0.53	39.9^b^ ± 0.63	<0.001
Weight (kg)	78.5^a^ ± 0.41	79.9^a^ ± 0.63	74.2^b^ ± 0.59	72.4^b^ ± 0.62	<0.001
Height (cm)	169.2^a^ ± 0.19	168.9^a^ ± 0.27	162.5^b^ ± 0.29	163.8^c^ ± 0.32	<0.001
BMI (kg/m^2^)	27.3^a^ ± 0.12	28.0^b^ ± 0.21	28.0^b^ ± 0.19	26.9^a^ ± 0.21	<0.001
FPG (mg/dl)	99.3^a^ ± 0.29	95.8^b^ ± 0.42	100.1^a^ ± 0.45	99.3^a^ ± 0.47	<0.001
OGTTT (mg/dl)	111.5^a^ ± 1.01	103.0^b^ ± 1.50	111.9^a^ ± 1.67	111.3^a^ ± 1.71	<0.001
HbA1C (%)	5.40^a^ ± 0.009	5.50^b^ ± 0.018	5.41^a^ ± 0.018	5.41^b^ ± 0.019	<0.001
Prevalence of prediabetes (%)					
OGT	7.3^a^	3.3^b^	6.6^c^	6.6^c^	<0.001
FPG	19.8^a^	12.9^b^	16.0^a^	16.6^a^	<0.001
HbA1C	20.0^a^	26.0^b^	20.0^a^	19.1^c^	<0.001

*^a–c^Values for continuous variables are in means ± SEs*.

*BMI, body mass index; FPG, fasting plasma glucose values 100–125 mg/dl; OGT, oral glucose tolerance test values 140–199 mg/dl; HbA1C, hemoglobin A1c values 5.7–6.4%; pairwise comparisons were done using, Tukey *post hoc* tests and chi-square tests for continuous and frequency variables, respectively, and values with different superscript differ at *P* < 0.05 levels; others, multiracial groups, and Hispanics*.

In Table 2, we compared trends in prevalence of prediabetes as defined by HbA1C, FPG, and OGT across age and BMI categories. Overall, HbA1C test was associated with a much higher prevalence of prediabetes in subjects who were over 50 years of age and subjects who were obese compared to using FPG or OGT test. Gradients of increasing prevalence of prediabetes from 12–19 to 70 years were observed for each blood glucose test (*P* < 0.01 for linear trend). Gradients of increasing prevalence of prediabetes across BMI categories of <20, 20–24.9, 25–29.9, 30–34.9, 35–39.9, and >40 kg/m^2^, representing low body weight, healthy weight, overweight, class I obesity, class II obesity and morbidly obese were also determined. As shown, gradients of increasing prevalence of prediabetes were evident using HbA1C, FPG, and IGT tests (*P* < 0.01 for linear trend).

**Table 2:** **Comparison of prevalence of prediabetes using HbA1C, FPG, and OGT tests in non-diabetic American children and adults**.

	HbA1C	FPG	OGT	*P*-value
**Age category (%)**
12–19	6.4	12.3	1.8	<0.001
20–29	5.8	12.3	2.2	<0.001
30–39	12.2	16.2	4.5	<0.001
40–49	21.2	20.3	7.9	<0.001
50–59	33.2	24.5	7.3	<0.001
60–69	38.1	24.4	10.2	<0.001
>70	38.8	23.2	12.9	<0.001
Test of linearity	<0.001	<0.001	<0.001	
**BMI category (%)**
<20	8.9	12.5	3.8	<0.001
20–24.9	14.8	15.5	4.2	<0.001
25–29.9	21.7^a^	21.8^a^	7.3	<0.001
30–34.9	29.6	21.7	9.7	<0.001
35–39.9	31.5	20.8	7.2	<0.001
>40	33.7	22.7	7.1	<0.001
Test of linearity	<0.001	<0.001	<0.001	

*^a^Values with same superscript did not differ at <0.05 level*.

*FPG, fasting plasma glucose; HbA1C, hemoglobin A1C; OGT, oral glucose tolerance test*.

### Correlation Between HbA1C, OGT, and FPG in Non-Diabetic Children and Adults

We examined race/ethnic-specific correlation between HbA1C and FPG, HbA1C and OGT, and FPG and OGT stratified by age and BMI categories (Table 3). The greatest degrees of correlation between HbA1C and FPG and between HbA1C and OGT were observed among Mexican-Americans who were over 69 years of age and with BMI of 40 or greater. A gradient of increasing correlation between HbA1C and FPG with increasing age was evident in the four racial/ethnic groups that were investigated, and ranged from 0.251 to 0.407, 0.148 to 0.463, 0.387 to 0.612, and 0.293 to 0.507 in non-Hispanic Whites, non-Hispanic Blacks, Mexican-Americans, and other races, respectively. A gradient of increasing correlation between HbA1C and OGT with increasing age was also evident in the four racial/ethnic groups that were investigated, and ranged from 0.126 to 0.334, 0.204 to 0.236, 0.128 to 0.606, and 0.183 to 0.444 in non-Hispanic Whites, non-Hispanic Blacks, Mexican-Americans, and other races, respectively.

**Table 3:** **Correlation between HbA1C and FPG, OGT and HbA1C, and OGT and FPG in non-diabetic American children and adults**.

	HbA1C and FPG				OGT and HbA1C				OGT and FPG			
	NHW	NHB	MA	OR	NHW	NHB	MA	OR	NHW	NHB	MA	OR
**Age category**												
12–19	0.251**	0.148	0.387**	0.293**	0.126	0.204	0.128	0.183	0.222*	0.377**	0.308**	0.133
20–29	0.207*	0.162	0.363**	0.392**	0.173	0.159	0.133	0.140	0.230*	0.301*	0.352**	0.251
30–39	0.330**	0.366**	0.169	0.293*	0.318*	0.050	0.275*	0.324*	0.472**	0.126	0.106	0.425**
40–49	0.214**	0.375**	0.236	0.329*	0.226**	0.054	0.063	0.076	0.424**	0.305*	0.043	0.328*
50–59	0.325**	0.377**	0.316*	0.249**	0.250**	0.137*	0.235**	0.210*	0.426**	0.188	0.178	0.334*
60–69	0.385*	0.427**	0.468**	0.456**	0.290**	0.210*	0.395**	0.324**	0.220*	0.255	0.223	0.020
>70	0.407**	0.463	0.612**	0.507**	0.334**	0.236**	0.606*	0.444**	0.246**	0.004	0.725*	0.398*
**BMI category**												
<20	0.241*	0.183	0.230	0.376**	0.147	0.153	0.248	0.115	0.315**	0.302	0.124	0.071
20–24.9	0.315**	0.209**	0.283**	0.338**	0.262**	0.194*	0.222*	0.259*	0.396**	0.341**	0.175	0.384**
25–29.9	0.236**	0.227**	0.291*	0.350**	0.232**	0.224**	0.110	0.352**	0.403**	0.367**	0.330**	0.473**
30–34.9	0.290**	0.189*	0.336**	0.365**	0.357**	0.235*	0.249*	0.337**	0.423**	0.249*	0.091	0.337**
35–39.9	0.385**	0.346*	0.462**	0.347*	0.371**	0.287**	0.285*	0.348*	0.482**	0.534**	0.385*	0.584*
>40	0.605**	0.457*	0.467**	0.435**	0.394**	0.388*	0.398**	0.374**	0.200	0.016	0.535	0.018

*NHW, non-Hispanic White; NHB, non-Hispanic Black; MA, Mexican-American; OR, other races; values are Pearson’s product-moment correlation coefficients*.

*FPG, fasting plasma glucose; HbA1C, hemoglobin A1C; OGT, oral glucose tolerance test*.

*P < 0.05; **P < 0.01.

A gradient of increasing correlation between HbA1C and FPG with increasing BMI was observed in the four racial/ethnic groups that were investigated, and ranged from 0.241 to 0.605, 0.183 to 0.457, 0.230 to 0.467, and 0.376 to 0.435 in non-Hispanic Whites, non-Hispanic Blacks, Mexican-Americans, and other races, respectively. A gradient of increasing correlation between HbA1C and OGT with increasing BMI was also seen in each racial/ethnic group that was investigated, and ranged from 0.147 to 0.374, 0.153 to 0.338, 0.248 to 0.398, and 0.115 to 0.374 in non-Hispanic White, non-Hispanic Black, Mexican-Americans, and other races, respectively. For FPG and OGT, the highest degrees of correlation were found in non-Hispanic Whites and other racial groups who were 30 to 59 years old, and non-Hispanic Blacks and Mexican-Americans who were 12–19 and over 70 years of age, respectively.

### Overall and Sex-Specific Prevalence of Prediabetes in Non-Diabetic American Children and Adults

**Figure 1:**
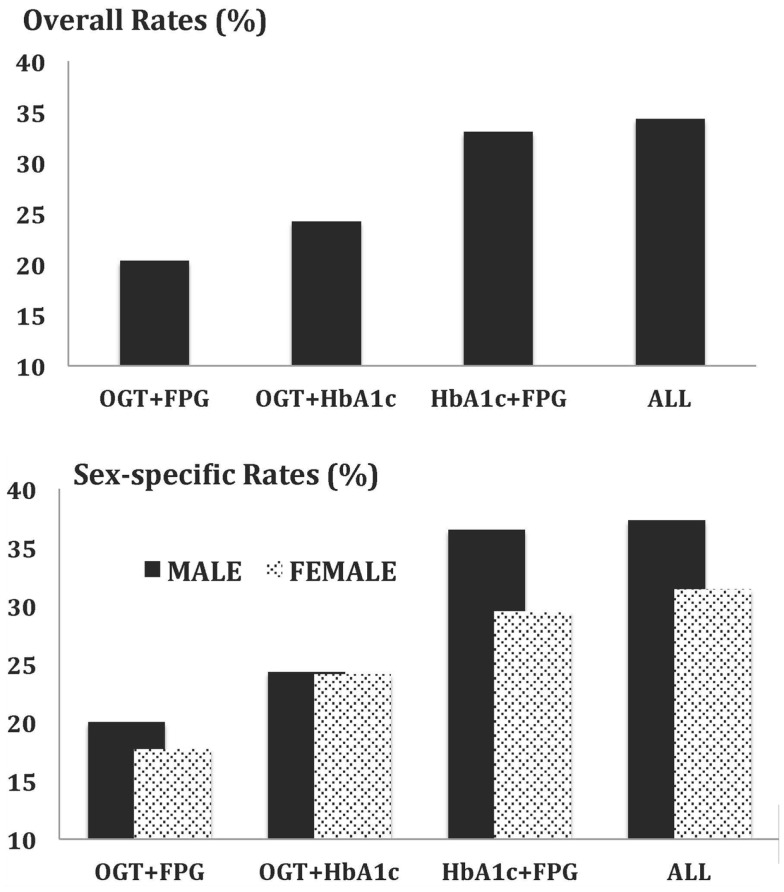
**Overall and sex-specific prevalence of prediabetes in non-diabetic American children and adults based on joint applications of blood glucose tests**.

Overall and sex-specific prevalences of prediabetes were assessed using various combinations of blood glucose tests that included OGT + FPG, OGT + HbA1C, HbA1C + FPG, and OGT + FPG + HbA1C. As shown in Figure 1, overall prevalences of prediabetes were 20.3, 24.2, 33, and 34.3% using OGT + FPG, OGT + HbA1C, HbA1C + FPG, and OGT + FPG + HbA1C, respectively. With the exception of OGT + HbA1C determined prevalence of prediabetes, overall prevalences of prediabetes were higher in male participants compared with females (*P* < 0.001). Results of comparative analysis indicated no statistically significant differences in overall and sex-specific prevalences that were determined using HbA1C + FPG and OGT + FPG + HbA1C tests (*P* > 0.05).

### Race/Ethnic and Sex-Specific Prevalence of Prediabetes in Non-Diabetic American Children and Adults

We compared prevalences of prediabetes using various combinations of blood glucose tests across race/ethnicity (Table 4). There were statistically significant racial/ethnic differences in prevalence of prediabetes using OGT + FPG, OGT + HbA1C, HbA1C + FPG, and OGT + FPG + HbA1C (*P* < 0.05). No statistically significant differences were observed for the prevalence of prediabetes using HbA1C + FPG and OGT + FPG + HbA1C tests. We compared prevalence of prediabetes for the traditionally used OGT + FPG test against HbA1C + FPG test. In men and women, HbA1C + FPG test resulted in a significantly higher prevalence of prediabetes compared with OGT + FPG test. In men, absolute differences between prediabetes prevalences determined using OGT + FPG and HbA1C + FPG tests were 11.6, 21.6, 9.5, and 8.4 in non-Hispanic Whites, non-Hispanic Blacks, Mexican-Americans and other racial/ethnic groups, respectively. The corresponding values in women were 12.4, 17.8, 7.4, and 8.3, respectively.

**Table 4:** **Race/ethnic-specific prevalence of prediabetes in non-diabetic American children and adults**.

Blood glucose tests	Non-Hispanic Whites	Non-Hispanic Blacks	Mexican-American	Others
**Overall**
OGT and FBG	21.8	14.3	22.3	21.4
OGT and HbA1C	24.5	27.4	21.1	22.8
HbA1C and FBG	34.0	33.7	30.8	31.8
OGT, FBG, and HbA1c	35.4	34.3	32.6	33.6
**Male**
OGT and FBG	25.9	16.0	24.6	23.4
OGT and HbA1C	23.6	30.6	19.7	22.8
HbA1C and FBG	37.5	37.0	34.1	31.8
OGT, FBG, and HbA1C	38.4	37.6	34.5	33.6
**Female**
OGT and FBG	18.3	12.7	19.9	19.5
OGT and HbA1C	25.5	24.2	22.6	22.2
HbA1C and FBG	30.7	30.5	27.3	27.8
OGT, FBG and HbA1C	32.4	31.0	30.7	30.3

*Other races, Hispanics, and multiracial groups*.

*FPG, fasting plasma glucose; HbA1C, hemoglobin A1C; OGT, oral glucose tolerance test*.

### Age-Specific Prevalence of Prediabetes in Non-Diabetic American Children and Adults

In Table 5, we compared prevalences of prediabetes using OGT + FPG, OGT + HbA1C, HbA1C + FPG, and OGT + FPG + HbA1C tests across age categories of 12–19, 20–29, 30–39, 40–49, 50–59, 60–69, and >70. Overall, gradients of increasing prevalences of prediabetes from 12–19 to >70 were evident using OGT + FPG, OGT + HbA1C, HbA1C + FPG, and OGT + FPG + HbA1C tests (*P* < 0.05 for linear trend). A gradient of increasing prevalence of prediabetes with increasing age was also seen in both men and women using OGT + FPG, OGT + HbA1C, HbA1C + FPG, and OGT + FPG + HbA1C tests (*P* < 0.05 for linear trend). Prevalences of prediabetes were higher using HbA1C + FPG test compared to using OGT + FPG test in every age group (*P* < 0.05). Prevalences of prediabetes were higher in men irrespective of the combinations of blood glucose tests that were used compared to women (*P* < 0.05). No statistically significant differences in prevalences of prediabetes were found using HbA1C + FPG and OGT + FPG + HbA1C tests. In men, the highest absolute difference in prediabetes prevalence between using HbA1C + FPG test and OGT + FPG test was 22.2 and was recoded in the >70 age group. The corresponding value in women was 27.5 and was observed in the 60–69-year-old group.

**Table 5:** **Age-specific prevalence of prediabetes in non-diabetic American children and adults**.

Blood glucosetests	Age categories (years)							
	12–19	20–29	30–39	40–49	50–59	60–69	70+	Total
**Overall**
OGT and FBG	13.2	13.4	17.6	23.1	26.1	27.8	26.9	20.3
OGT and HbA1C	7.8	7.9	15.5	27.4	37.3	43.5	45.1	24.2
HbA1C and FBG	17.5	16.8	25.2	36.8	47.4	51.3	50.3	33.0
OGT, FBG, and HbA1C	18.2	17.7	26.2	39.0	48.4	53.3	52.4	34.3
**Male**
OGT and FBG	17.5	17.7	20.1	23.8	28.7	29.9	29.2	23.0
OGT and HbA1C	9.2	9.4	16.5	30.2	37.3	38.7	48.4	24.3
HbA1c and FBG	2.5	23.4	30.6	42.3	50.7	49.0	51.4	36.5
OGT, FBG, and HbA1C	23.2	23.6	30.8	42.8	51.0	50.4	53.7	32.3
**Female**
OGT and FBG	8.6	8.9	15.3	22.4	23.6	25.9	24.8	17.6
OGT and HbA1c	6.2	6.4	14.6	24.8	37.4	47.9	45.8	24.1
HbA1c and FBG	12.0	10.2	20.0	31.8	46.2	53.4	49.3	25.5
OGT, FBG, and HbA1C	12.7	11.8	21.6	35.6	45.7	55.9	51.2	31.4

*FPG, fasting plasma glucose; HbA1C, hemoglobin A1C; OGT, oral glucose tolerance test*.

### BMI-Specific Prevalence of Prediabetes in Non-Diabetic American Children and Adults

In Table 6, we compared prevalences of prediabetes across BMI categories of <20, 20–24.9, 25–29.9, 30–34.9, 35–39.9, and >40, representing low body weight, healthy weight, overweight, class I obesity, class II obesity and morbidly obese, respectively. Overall, gradients of increasing prevalence of prediabetes from low body weight to morbidly obese were evident using OGT + FPG, OGT + HbA1C, HbA1C + FPG, and OGT + FPG + HbA1C tests (*P* < 0.05 for linear trend). A gradient of increasing prevalence of prediabetes with increasing BMI was also found in both men and women (*P* < 0.05 for linear trend). Results of comparative analysis of prediabetes prevalence values indicated no differences in overall and sex-specific prevalences at each level of BMI categories using HbA1C + FPG and OGT + FPG + HbA1C tests (*P* > 0.05).

**Table 6:** **BMI-specific prevalence of prediabetes in non-diabetic American children and adults**.

Blood glucosetests	Body mass index categories (kg/m^2^)						
	<20	20–24.9	25–29.9	30–34.9	35–39.9	40+	Total
**Overall**
OGT and FBG	14.5	17.4	23.9	24.5	22.2	23.9	21.1
OGT and HbA1C	11.6	17.7	26.1	35.3	36.5	36.9	25.2
HbA1C and FBG	19.8	26.4	36.9	43.5	44.4	46.3	34.3
OGT, FBG, and HbA1C	21.4	27.9	38.3	45.0	45.6	47.1	35.8
**Male**
OGT and FBG	18.7	22.0	26.9	23.4	21.8	27.7	23.9
OGT and HbA1C	12.7	18.8	25.4	34.5	36.1	39.1	25.1
HbA1C and FBG	23.9	31.5	40.3	45.5	46.3	49.5	37.9
OGT, FBG, and HbA1C	26.3	32.6	41.0	45.5	47.6	49.5	38.7
**Female**
OGT and FBG	11.0	13.0	20.4	25.6	22.4	21.4	18.4
OGT and HbA1C	10.7	16.7	27.0	36.1	36.8	35.1	25.2
HbA1c and FBG	16.3	21.4	32.8	41.5	43.4	44.2	30.9
OGT, FBG, and HbA1C	17.3	23.2	35.0	44.4	44.5	45.5	32.8

*FPG, fasting plasma glucose; HbA1C, hemoglobin A1C; OGT, oral glucose tolerance test*.

## Discussion

While each of the blood glucose tests has its advantages, there is no consensus on the best and the most appropriate blood glucose test for screening for prediabetes in clinical and population-based settings. In clinical practice, the OGT test is the gold standard diagnostic test for type 2 diabetes. FPG testing alone lack enough sensitivity to diagnose type 2 diabetes (4, 27). Indeed, diagnosis of hyperglycemia using only FPG testing without including OGT testing can lead to the omission of a large number of subjects with normal FPG who have elevated OGT results. In population-based settings, a screening test that lessens problems of OGT test may yield a better prevalence estimate of prediabetes. HbA1C appears to be a good test for improving the low yield associated with FPG test while eliminating the need for fasting or waiting 2 h following ingestion of a glucose load associated with OGT testing.

We argue that for community-based population screening, HbA1C test is better than other blood glucose tests or at least as good as both FPG and OGT tests for the reasons mentioned above. In the field, HbA1C test can be performed with a point of care (POC) device that offers the following advantages: study participants do not need to be fasting, testing is not effected by content and timing of previous meals, and results are immediately available. For both community-based research and for community-based screening, this offers the opportunity for immediate discussion and, if necessary, referral for further testing and care if necessary. Indeed, in screening for prediabetes, HbA1C test has been shown to be easier for patients and health providers particularly during patient health care interactions regardless of patient health conditions (28). Furthermore, HbA1C assay is an excellent technique in identifying people who are at risk for diabetes complications who could benefit from validated treatments (9, 29). In addition, HbA1C is excellent for predicting cardiovascular outcomes (8). Notwithstanding the advantages of these blood glucose tests, screening for prediabetes using each alone or using a combination of FPG and OGT often produces dissimilar estimates (29, 30). Hence, it has been hinted that each blood glucose test evaluates different domains of blood glucose homeostasis (29, 31, 32). Indeed, damage in insulin secretion has been found to be more germane in IFG, whereas unsteady insulin sensitivity is exceptional to IGT (29, 31). Since no one blood glucose test is able to identify all subjects with prediabetes, determining a combination of blood glucose tests that is associated with identification of the greatest number of subjects with prediabetes is critical for diabetes prevention programs (DPPs). To the best of our knowledge, very few studies (32, 33) have examined whether combined use of blood glucose tests in screening will enhance detection of prediabetes in populations at risk for diabetes. Therefore, this study was designed to determine the combination of blood glucose tests that is associated with highest prevalence of prediabetes across age, sex, race/ethnicity, and BMI categories.

In this study, correlation of HbA1C with FPG and OGT were higher in Mexican-Americans who were 60 years old or greater compared to other racial/ethnic groups. Additional studies are needed to clarify reasons for the observed racial/ethnic differences in HbA1C values as well as the observed concordance among blood glucose tests. In this study, ADA criteria for HbA1C resulted in much higher prevalences of undiagnosed prediabetes than were estimated from FPG and OGT tests. Prevalences of prediabetes using OGT tests were 7.3, 3.3, 6.6, and 6.7% in non-Hispanic Whites, non-Hispanic Blacks, Mexican-Americans, and other racial/ethnic groups, respectively. Prevalence values of prediabetes using FPG tests were 19.8, 12.9, 16, and 16.6% in non-Hispanic Whites, non-Hispanic Blacks, Mexican-Americans, and other racial/ethnic groups, respectively. The corresponding values using HbA1C tests were 20, 26, 20, and 19.1%, respectively.

Joint use of FPG and HbA1C test was associated with significantly higher proportion of subjects with prediabetes across age, race/ethnicity and BMI as compared to combined use of OGT and FPG, OGT and HbA1C. Overall prevalences of prediabetes using OGT + FPG, OGT + HbA1C, HbA1C + FPG, and OGT + FPG + HbA1C tests were 20.3, 24.2, 33, and 34.3%, respectively. This study indicates prediabetes rates were higher using HbA1C + FPG tests compared to the traditionally used OGT + FPG tests. Compared to OGT + FPG test, the use of HbA1C + FPG test in screening was associated with 44.8, 135, 38.6, and 35.9% increased prevalence of prediabetes in non-Hispanic White, non-Hispanic Black, Mexican-American, and other racial/ethnic men, respectively. The analogous values in women were 67.8, 140, 37.2, and 42.6%, respectively. Overall, gradients of increasing prevalence of prediabetes from 12–19 to 70 years and greater were evident using OGT + FPG, OGT + HbA1C, HbA1C + FPG, and OGT + FPG + HbA1C tests. In men and women, prevalences of prediabetes were also found to increase linearly with increasing BMI. Using all blood glucose tests (OGT + FPG + HbA1C) did not improve the overall and sex-specific prediabetes prevalence beyond what was observed using HbA1C + FPG test.

Our result, indicating higher prevalence of prediabetes using FPG test compared with OGT test, is consistent with findings by Unwin et al. (34) who compared glycemic criteria in three ethnic groups in the UK. The results of this study, implying differences in the prevalence of prediabetes using HbA1C, OGT, and FPG, suggest that these blood glucose tests may be measuring different aspects of blood glucose metabolism. The finding of a far greater prevalence of prediabetes in non-Hispanic Blacks using HbA1C criteria as compared with using OGT and FPG tests is in agreement with findings from the DPP. In DPP study, non-Hispanic Blacks with IGT were also found with much elevated HbA1C levels compared to non-Hispanic Whites, upon adjusting for glucose levels, age, sex, education, blood pressure, BMI, hematocrit, and insulin resistance (16). Our finding of greater prevalence of prediabetes in non-Hispanic Blacks using HbA1C criteria as compared with using OGT and FPG is unclear and may not be related to blood glucose control factors and our observed racial/ethnic differences in the prevalence of prediabetes using HbA1C test may be associated with to racial/ethnic differences in hemoglobin glycation or red cell survival (11, 12). The reported within individual variation in HbA1C at the same levels of glycemia (11, 12, 16) may be explained by racial/ethnic differences in red cell permeation, glycation, red cell half-life, vitamin and medication use (11, 12).

The main strength of this report is in the use data from the NHANES. NHANES represents one of the best available data sources because it is a representative U.S. data. The measurements and assays in NHANES were done using consistent techniques. The training and quality control measures of NHANES give added reliability to the data. However, the use of a single determination of each of the blood glucose tests is a significant limitation of this study. The use of a single glycemic measure is often associated with overestimation of undiagnosed prediabetes prevalence (12).

## Conclusion

Using a combination of HbA1C and FPG test in screening for prediabetes provides the benefits of individual test and decreases the risk of systematic bias inherent in using only HbA1C testing. This study proposes the need to redefine a basic and a practical way on the application of HbA1C in screening for prediabetes. The use of HbA1C test in detection of prediabetes in population and clinical settings should take into account patient race/ethnicity, gender, age, and BMI.

## Conflict of Interest Statement

The authors declare that the research was conducted in the absence of any commercial or financial relationships that could be construed as a potential conflict of interest.
